# A TNF-induced gene expression program under oscillatory NF-κB control

**DOI:** 10.1186/1471-2164-6-137

**Published:** 2005-09-28

**Authors:** Bing Tian, David E Nowak, Allan R Brasier

**Affiliations:** 1 Department of Medicine, The University of Texas Medical Branch, 301 University Blvd., Galveston, Texas 77555-1060, USA; 2 Sealy Center for Molecular Sciences, The University of Texas Medical Branch, 301 University Blvd., Galveston, Texas 77555-1060, USA

## Abstract

**Background:**

The cytokine tumor necrosis factor (TNF) initiates tissue inflammation, a process mediated by the NF-κB transcription factor. In response to TNF, latent cytoplasmic NF-κB is activated, enters the nucleus, and induces expression of inflammatory and anti-apoptotic gene expression programs. Recently it has been shown that NF-κB displays two distinct activation modes, monophasic and oscillatory, depending on stimulus duration. Characterization of temporal expression patterns for the NF-κB network and determination of those genes under monophasic- or oscillatory control has not been experimentally addressed.

**Results:**

To identify the kinetics of NF-κB-dependent gene expression and determine whether these two types of NF-κB translocation modes control distinct gene programs, a detailed kinetic analysis of a validated microarray data set was performed on 74 unique NF-κB-dependent genes in response to TNF. Hierarchical clustering identified distinct expression profiles termed the "Early", "Middle", "Late" response groups, peaking 1, 3, and 6 h after stimulation, respectively. These expression patterns were validated by Quantitative Real Time PCR (Q-RT-PCR) and NF-κB binding was demonstrated by chromatin immunoprecipitation (ChIP) assays. Each response group was mapped to its molecular function; this analysis indicated that the Early group encodes cytokines or negative regulators of the IKK-NF-κB pathway, and the Late group encodes cell surface receptors, adhesion molecules and signal adapters. That similar coordinated sequential cascades of gene expression were also seen in response to stimulation by the cytokine IL-1, and expression patterns observed in MRC-5 fibroblasts indicated that the epithelial NF-κB program is relatively stimulus- and cell type-independent. Bioinformatic analysis of the Early and Late gene promoters indicates that although both groups contain similar patterns of NF-κB-binding sites, only the Early gene promoters contain NF-κB-binding sites located in phylogenetically conserved domains. Stimulation protocols designed to produce either monophasic or oscillatory NF-κB activation modes showed that the oscillatory mode is required only for expression of the Late genes.

**Conclusion:**

This analysis provides important insights into the TNF-regulated genetic response program in epithelial cells, where NF-κB controls sequential expression patterns of functionally distinct genes that depend on its oscillatory activation mode.

## Background

Tumor necrosis factor (TNFα, TNF ligand superfamily 2 [TNFSF2]) is a prototypical inflammatory and immunomodulatory cytokine inducibly expressed by activated macrophages, monocytes, neutrophils, T-cells and NK-cells [1]. TNFα is a central mediator of the host inflammatory response by its ability to activate adhesion molecule expression, enhance leukocyte trafficking, and affect the expression of secondary cytokine cascades controlling leukocyte recruitment and activation [1,2]. TNF signaling is mediated by binding and aggregating single-pass type I transmembrane receptors (TNFR-I, ref. [3]) that then serve as an anchor to recruit signaling proteins binding to the death domains on the cytoplasmic receptor tails. Upon assembly of this submembranous complex, two major downstream signaling pathways are activated; these are the jun NH_2 _terminal kinase -activating protein-1- and the IκB Kinase (IKK)-Nuclear Factor-κB (NF-κB) pathways [4,5].

Nuclear Factor-κB (NF-κB) is a latent cytoplasmic transcription factor maintained in a cytoplasmic location by binding the IκB inhibitors, proteins that bind and specifically inactivate it by masking its nuclear localization sequence, thereby preventing its nuclear entry [6]. NF-κB is activated by TNF signaling pathway indirectly as a result of targeted IκB proteolysis (reviewed in ref. [7]). Signal-induced IκB proteolysis is mediated by activation of the multiprotein cytoplasmic IKK (a.k.a., the "signalsome"ref. [8]), a kinase that phosphorylates IκB specifically in its NH_2_-regulatory domain, making it a substrate for proteolysis through the 26S proteasome and calpain pathways [8,9].

As a result, liberated NF-κB rapidly enters the nucleus to activate target gene expression by formating a nucleoprotein complex with chromatin-remodeling proteins, kinases, and other transcription factors [10]. Recent single cell fluorescence imaging experiments have shown that TNF can induce two distinct modes of NF-κB activation patterns [11]. In the monophasic mode, the result of a brief TNF stimulation, NF-κB enters the nucleus and induces the expression of IκB inhibitory proteins whose resynthesis redistributes NF-κB back into the cytoplasm, restoring cellular homeostasis [12,13]. Conversely, oscillatory NF-κB activation, a result of tonic TNF stimulation produces prolonged IKK activation and continued IκB proteolysis, results in repeated rounds of NF-κB translocation and cytoplasmic recapture [11]. This latter activation profile is characterized by a series of asynchronous, damped oscillations of nuclear NF-κB [14]. These findings explain the biphasic pattern of nuclear NF-κB binding that has been observed in response to tonic TNF stimulation in a number of distinct cell types [15,16], where the initial oscillation is observed due to stimulus-induced synchrony in the cell population, but lost afterwards because subsequent oscillations are asynchronous and appear damped in the population [14]. Whether these two modes of NF-κB activation produce distinct genetic programs has not been systematically studied.

Although a body of isolated work has reported that NF-κB controls expression of acute-phase reactants [17], cytokines [18], anti-apoptotic proteins [19], and autoregulators of the IKK-NF-κB pathway [12,13,20], the full spectrum of NF-κB-dependent genes are only beginning to be systematically understood [21]. In this study, we analyzed and validated a microarray time series experiment of cells expressing a regulated NF-κB dominant-negative inhibitor in response to TNF. From this data set, we have previously systematically identified known and novel NF-κB-dependent genes [21]. Because these represent a time series experiment, the data may contain genes that are under direct or indirect NF-κB control. That these "NF-κB-dependent" genes were *directly *controlled by NF-κB was verified by satisfying a series of experimental validation experiments: 1. Ectopic expression of constitutively active NF-κB/Rel A transactivated the endogenous "NF-κB-dependent" genes in the absence of TNF stimulation; 2. TNF induced the "NF-κB-dependent" genes in the absence of new protein synthesis; 3. NF-κB sites were computationally identified and confirmed by EMSA; and, 4. Chromatin immunoprecipitation (ChIP) assays showed the endogenous "NF-κB-dependent" promoters bound NF-κB/Rel A in TNF stimulated cells [21]. Based on these observations, we concluded that this was a robust dataset containing genes directly under NF-κB control.

Here we performed a kinetic analysis of the time series data set where, strikingly, four distinct kinetic groups were identified by cluster analysis. Gene Ontology and Ingenuity pathway analysis show that these response groups encode distinct biological functions from one another, with the Early group being composed of families of secreted cytokine/chemokines and the Late group being composed of cell surface receptors and adhesion molecules. Stimulation experiments producing monophasic or oscillatory NF-κB activation modes show that the oscillatory mode is required for Late gene expression. These data provide major new insights into the coordinated NF-κB response program to inflammatory stimuli, where the cellular response is dictated by the mode in which NF-κB is activated.

## Results

**Figure 1:**
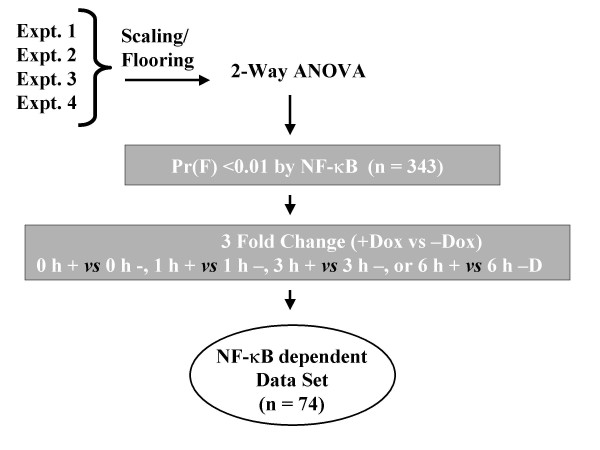
Schematic diagram of microarray data analysis. HeLa^TetO-FLAG-IκBα Mut ^cells were plated in parallel into cultures in the absence or presence of Dox (2 μg/ml). After four days, cells were stimulated without (0 h) or with rhTNFα (25 ng/ml) at 6 h, 3 h, and 1 h prior to simultaneous harvest for RNA extraction. Experiments were conducted four independent times. Data sets were scaled for comparison. NF-κB dependent genes were identified using 2-way ANOVA where Dox treatment and TNF treatment were considered independent variables. Those changed by Dox treatment at a p-values [Pr(F)< 0.01] were then filtered for 3-fold change at any point during the experiment (signal intensity with NF-κB vs signal intensity without NF-κB).

**Figure 2:**
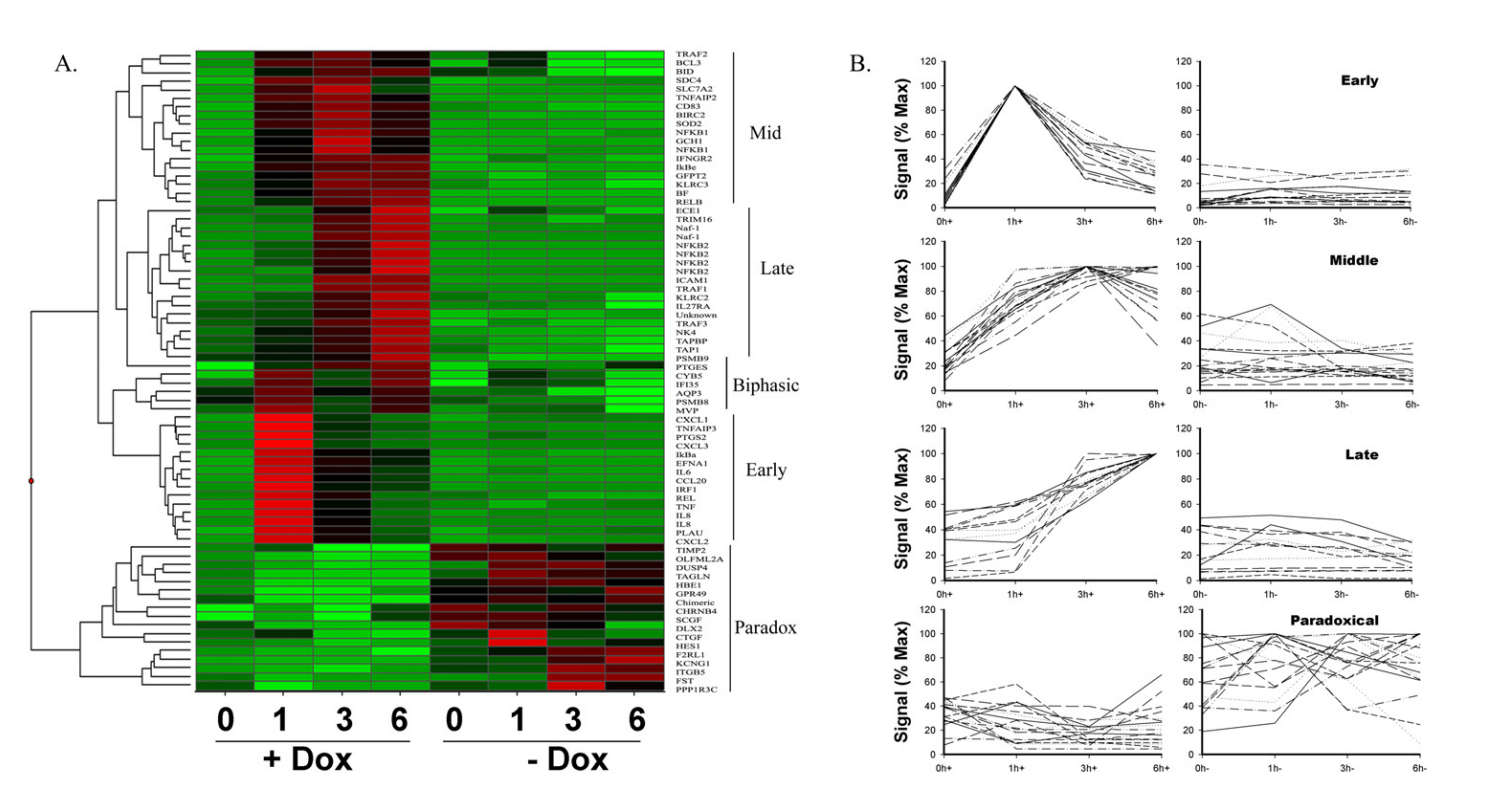
Temporal Cascades of NF-κB Regulated Genes. **(a) **The Signal Intensity values from 74 probe sets identified as being NF-κB dependent were Z-score normalized and subjected to hierarchical clustering. Red corresponds Z > +2.5, green indicates Z < 0, and black indicates Z > 0.5. Expression groups are indicated at right by vertical line. **(b) **Distinct Expression Profiles. The normalized SI measurements for each of the genes in Clusters I–IV are presented as a percentage of the maximum value for any point across the stimulation.

We have previously isolated and characterized HeLa^tTA/FLAG-IκBα Mut^, a clonal cell line expressing a tetracycline-regulated NF-κB dominant-negative inhibitor [21,22]. When doxycycline (Dox) is present in tissue culture medium, tTA is inactivated, and FLAG-IκBα Mut levels are barely detectable by Western immunoblot, resulting in a wild type phenotype, with normal levels of activated NF-κB in the nucleus being produced after stimulation [21,22]. Conversely, upon Dox withdrawal, tTA is activated, and FLAG-IκBα Mut expression occurs at similar levels to endogenous IκBα [22]. These levels of FLAG-IκBα Mut expression are sufficient to completely inhibit NF-κB translocation and target gene expression [21,22]. HeLa^tTA/FLAG-IκBα Mut ^cells were plated in parallel cultures in the absence or presence of Dox (2 μg/ml), and each group stimulated tonically with TNFα to induce NF-κB activation in the oscillatory mode [11]. RNA was then subjected to high density oligonucleotide microarray analysis. Reanalysis of the raw data set was performed using less stringent statistical filters to more fully identify the spectrum of biological functions under NF-κB control, where we identified 74 probe sets (Figure 1). From these, the scaled signal intensities were subjected to hierarchical clustering to identify coregulated genes, identifying 5 expression groups based on their kinetics of expression (Figure 2a). As an indicator of reproducibility, we noted that redundant probe sets generally clustered with one another, where multiple NF-κB2 and IL-8 probesets group together (Figure 2a). These findings indicated that the clustering analysis is robust, grouping probe sets representing the same genes based on similar expression patterns. Further inspection of the hierarchical clustering results indicates that TNF induces expression of five distinct groups: 1. "Early" genes whose expression profiles peak at 1 h and less; 2. "Middle" genes whose expression profiles peak at 3 h, falling thereafter; 3. "Late" genes whose expression profiles begin to peak at 6 h and later; 4. "Biphasic", genes whose expression profiles peak rapidly at 1 h, fall at 3 h, and peak again at 6 h; and, 5. "Paradoxical" genes whose expression is not significantly altered by TNF in the wild type cells, but whose expression are induced by TNF in the cells lacking NF-κB signaling (Figure 2a). Further, NF-κB dependence for these probe sets is seen by comparing the heat map profiles for each probe set obtained in the presence of Dox vs the profile obtained in the absence of Dox (Figure 2a). For example, the strong induction of gene expression in "Middle" genes at 3 h in the presence of Dox is not seen in the absence of Dox. Similar findings are made for the probe sets in the "Early", "Late", and "Biphasic" genes.

To rigorously compare the expression profiles of the major clusters, the scaled and normalized hybridization intensities were retrieved for each gene and plotted by group as a percentage of each gene's maximal expression value during the time course (because of the limited number of probe sets in the Biphasic group, these were excluded from subsequent analysis). As seen in Figure 2b, as a group, the Early genes had ~ 10 % maximal expression at time 0, and rapidly peaked at 100% maximal expression by 1 h, falling to ~ 50 % expression at 3 h. The Middle and Late groups tended to have higher basal expression relative to their maximal induction. The genes within the Late group were tightly synchronized showing maximal expression at 6 h. As expected, the Paradoxical group showed no significant induction by TNF in the presence of Dox, but their expression increased 2-fold in the cells stimulated in its absence. As a test whether the TNF-induced profile grouping was statistically significant, the paired two-tailed Z-Test statistic was calculated to determine whether these three expression groups came from different populations during the TNF response [see Additional file 1]. We found that the P values for the two-tailed test statistic for Early gene group is significantly different than the profiles of the Middle or Late genes at all times of TNF stimulation, indicating that they come from a distinct population than the members of the Middle or Late genes. Similarly, the expression profiles of the Middle genes are different from the Late genes also at 1, 3, and 6 h of stimulation. Together this analysis indicated that the Early, Middle and Late expression groups are separable populations with distinct gene expression patterns.

**Figure 3:**
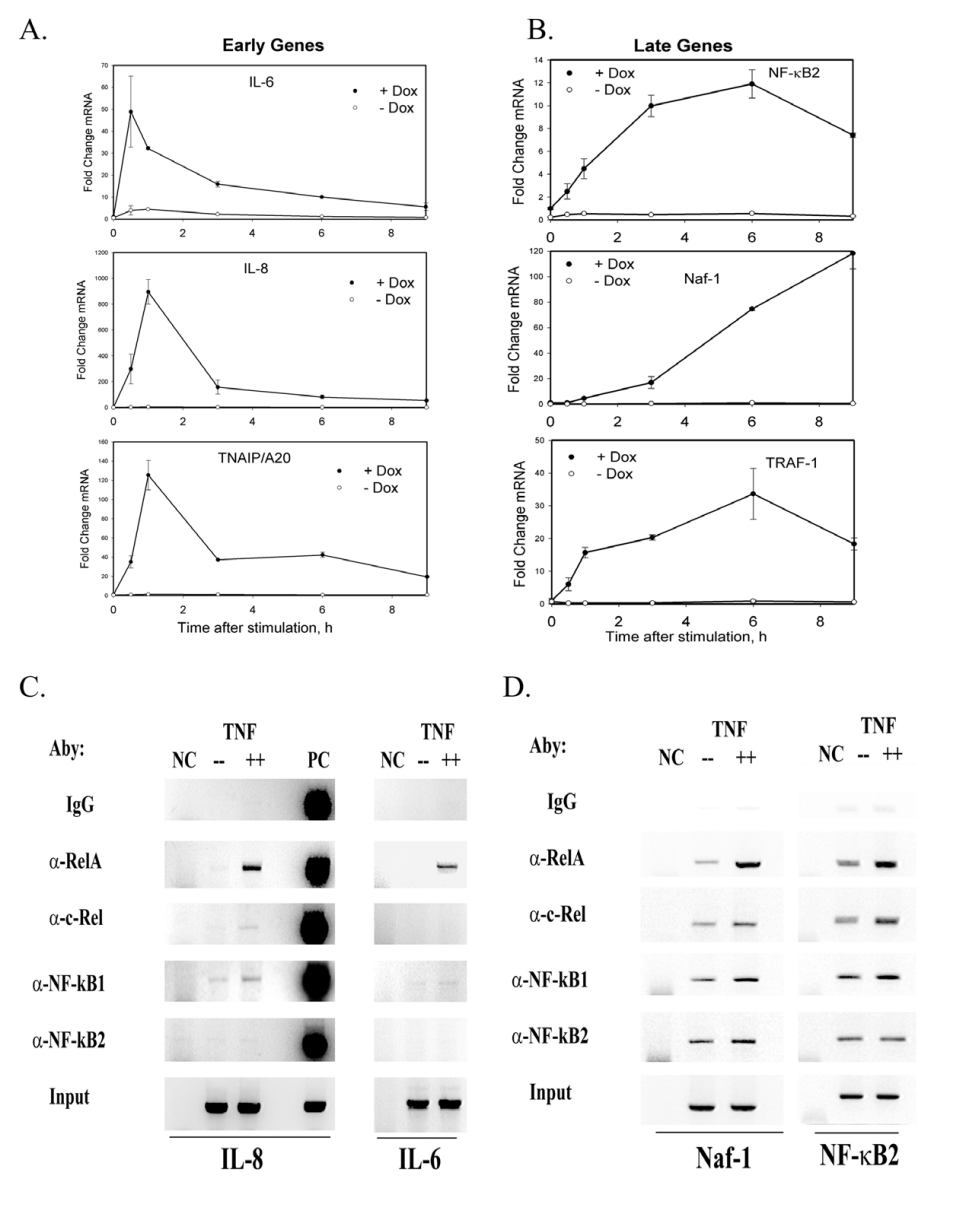
Validation of expression profiles and NF-κB dependence. **(a) **Early gene profiles. HeLa^tTA/FLAG-IκBα Mut ^cells were plated in parallel in the absence or presence of Dox (2 μg/ml) and stimulated with rhTNFα. Changes in mRNA abundance (normalized by 18S) determined by Q-RT- PCR from total RNA. For each of the indicated mRNA transcripts, values are expressed as fold change relative to unstimulated cells and plotted on a logarithmic scale. +/-Dox, data obtained from cells cultured with or without Dox. **(b) **Late gene profiles. Experiment and data analysis are as in Figure 3a. **(c) **ChIP for NF-κB subunit binding to Early Gene promoters. ChIP was performed on control or TNFα-stimulated (30 min, 20 ng/ml) HeLa cells using the antibodies indicated at left. Shown is an ethidium-bromide stained agarose gel of the PCR products performed under linear amplification conditions. The target gene is indicated at the bottom. NC, negative control reaction (no template is added to the PCR reaction); PC, positive control reaction (25 ng of genomic DNA is used as template in PCR). **(d) **ChIP for NF-κB subunit binding to Late Gene promoters. ChIP was performed on HeLa cells stimulated as in Figure 3c.

To validate the gene expression kinetics and confirm their NF-κB dependence, Q-RT-PCR assays were developed for representative members of the Early (IL-6, IL-8, TNFAIP3/A20) and Late (NF-κB-2, Naf-1 and TRAF-1) genes. We then used these assays to measure mRNA changes in tonic TNFα-stimulated in HeLa^tTA/FLAG-IκBα Mut ^cells cultured in the absence or presence of Dox. A rapid induction of mRNA transcripts was observed within 1 h for the selected Early genes (Figure 3a). Here, IL-6 mRNA abundance peaked at 48 -fold within 0.5 h, whereas IL-8 and TNFAIP3/A20 peaked at 900-fold and 125-fold at 1 h, respectively. All mRNA signals then rapidly fell to < 30% of the maximal signal at 3 h of stimulation (Figure 3a). In addition, in the HeLa^tTA/FLAG-IκBα Mut ^cells cultured in the absence of Dox, mRNA expression for all of these genes were significantly inhibited, with IL-6 being induced no more than 5- fold, and no detectable induction was seen for IL-8 and TNFAIP3/A20 (Figure 3a). In contrast, mRNA transcript abundance for the Late gene group peaked 6 h after TNFα stimulation, with the exception of Naf-1, whose mRNA abundance continued to increase until 9 h (Figure 3b). Like the Early genes, TNFα-induced expression of the Late genes was also significantly inhibited in cells cultured in the absence of Dox.

We next asked whether TNF-induced specific NF-κB subunit binding to endogenous target genes. For this purpose, Chromatin immunoprecipitation (ChIP) assays were performed on representative members of the Early (IL-8, IL-6) and Late (Naf-1, NF-κB2) genes. In this assay, control or TNF-stimulated cells are exposed to protein-DNA cross-linking reagents to covalently stabilize the chromatin. The soluble chromatin is then extracted, sheared, and the target protein specifically immuno-precipitated (along with its associated DNA). After elution of the DNA, the crosslinks are reversed, and the presence of specific genes detected by PCR. As seen in Figure 3c, TNFα treatment strongly induced NF-κB/RelA subunit binding the IL-8 gene. By contrast, TNF induced only weak c-Rel binding to IL-8. For the DNA binding subunits, although NF-κB1 binding is detectable on IL-8 in the absence of stimulation (compare signal in absence of TNF vs that produced by IgG, Figure 3c), its levels increase in response to TNF treatment. By contrast, no NF-κB2 binding is detectable either in the presence or absence of TNF stimulation. Very similar patterns of NF-κB subunit binding are seen by ChIP assay of the IL-6 gene (Figure 3c, right panel). In Figure 3d, basal- and TNF-induced binding of the same NF-κB subunits is shown for two representative members of the Late genes. Like the early genes, TNF induces RelA and to a lesser extent, c-Rel, and NF-κB1 binding to both the Naf-1 and NF-κB2 genes (Figure 3d). In contrast, constitutive NF-κB2 binding is seen for both promoters. Because of differences in PCR efficiencies, it is not possible to determine whether NF-κB2 subunits are binding more or less strongly to the Late gene promoters than the Early genes. These findings indicate that the selected Early and Late genes directly and inducibly bind NF-κB/RelA, c-Rel, and NF-κB1 DNA binding subunits in an apparently similar pattern. Taken together, these studies validate the microarray profiles, confirm the relative cascades of gene expression, demonstrate their absolute dependence on intact NF-κB signaling, and indicate the Early and Late members show similar binding affinities for the transactivating NF-κB family subunits.

**Figure 4:**
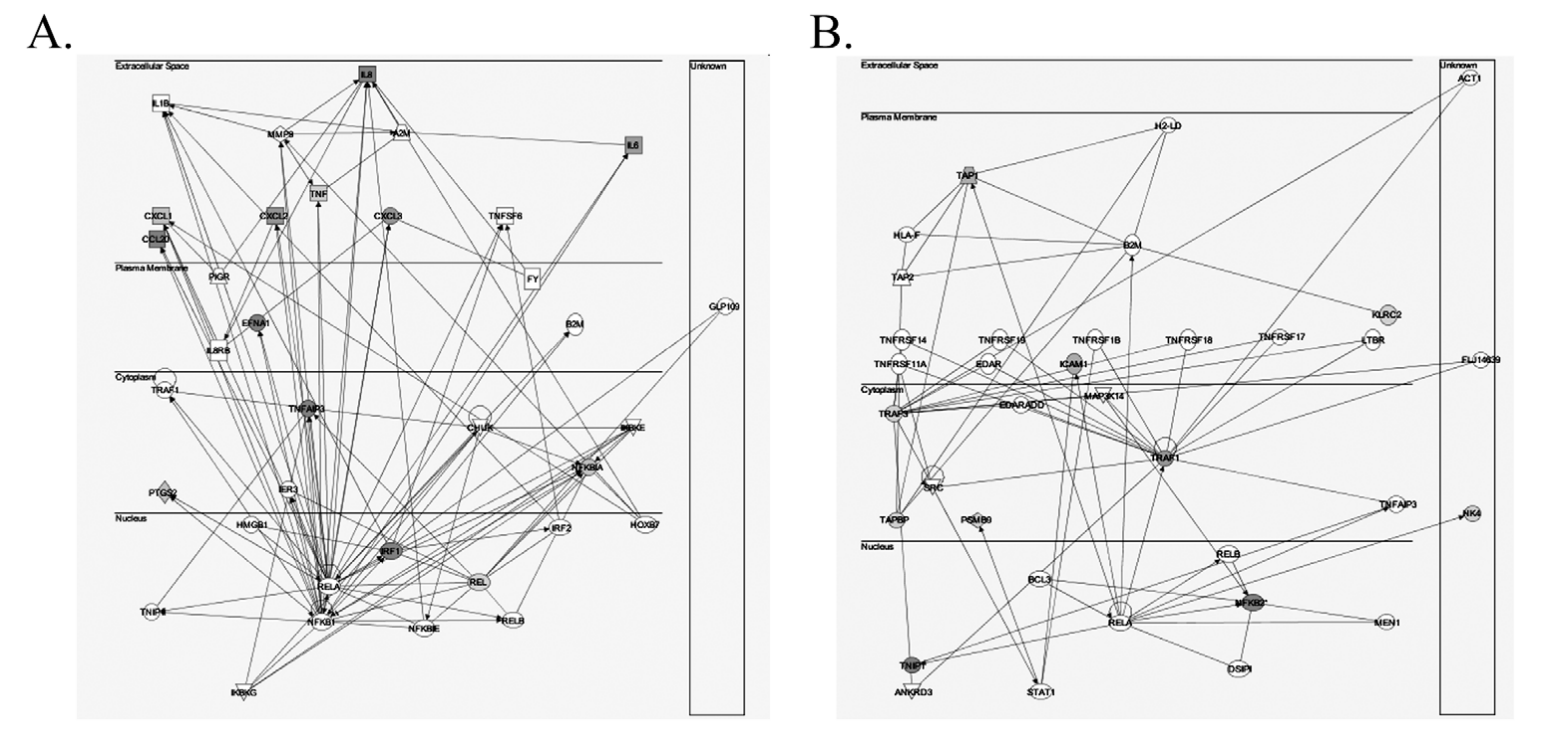
Ingenuity Pathway Analysis of biological pathways controlled by Early and Late genes. **(a) **Early gene pathway. Shown is a graphical representation of the highest scoring pathway controlled by the genes in Cluster III. Shown are labeled nodes representing individual protein functions and their relationship represented by edges. Nodes are colored by changes in expression, with red indicating > 10 fold change; pink > 2-fold and < = 10-fold change; no color indicating < = 2-fold change or data is not present. Squares indicate cytokines, circles indicate chemokines, and ovals indicate transcription factor. For the edges, an arrow indicates "acts on". Horizontal lines indicate the most likely subcellular location for the protein encoded by each node. See Legend to Table II for the index of relevant abbreviations. **(b) **Late gene pathway. Graphical representation of the highest scoring pathway controlled by the genes in Cluster III. See Fig. 2A for explanation of figure and symbols.

To determine the functional activities of the various NF-κB dependent groups, the individual probe sets in each expression profile genes were mapped to their primary Gene Ontology Biological Process and Molecular Function. Statistical analysis was performed on functional categories over-represented in the groups relative to the functional representation within the human proteome (Table 1, ref. [23]). For example, Cytokine Activity was significantly enriched in the Early dataset, representing 32 % of the genes, whereas peptide transporter and protein binding activity was enriched in the Late dataset, representing 12- and 31 % of the genes, respectively (Table 1). To more clearly display this functional difference, the 74 NF-κB-dependent genes were annotated by primary biochemical function and kinetic grouping (Table 2). From this analysis, it is clear that the NF-κB-dependent genes control a variety of cellular processes, including anti-apoptosis, cytokine signaling, growth factors and secreted proteins, metabolism, receptors and cell-surface adhesion molecules, signaling molecules, transcription factors and those with currently unknown function. Here, our analysis reveals that groups of genes controlling distinct cellular functions are sequentially expressed during the evolution of the TNF response. For example, the Early gene group is predominantly composed of secreted cytokines, including IL-6, IL-8, CXCL-1 through- 3, TNF and CCL20/Exodus-1 (Table 2). Conversely, the Late gene group encodes cell-surface adhesion molecules (ICAM, KLRC2), signaling adapter molecules (TRAF1/3), and NF-κB2. The Middle group functionally overlaps with those of the Late genes in that they control expression of cell-surface receptors, signaling molecules, autoregulators of the IKK-NF-κB pathway and metabolic enzymes. To avoid the potential problem of bias inherent in expert classification, we employed Ingenuity Pathways Analysis (IPA). IPA compares groups of genes from each expression profile to an annotated database generated from published protein and genetic networks and displays a rank-ordered list of pathways whose function is most likely to be affected by that expression pattern. For each pathway, its members and their relationships (functional and physical) are displayed. Consistently, the highest scoring IPA pathway for Early gene group was an NF-κB-dependent pathway controlling production of extracellular cytokines (Figure 4a). Similarly, although the highest scoring pathway for Late gene group was also an NF-κB-dependent pathway, the major targets of this pathway are extracellular adhesion proteins (Figure 4b). Together, our data suggests that NF-κB controls waves of sequential expression of functionally distinct genes.

**Table 1:** GO Mapping of NF-κB-dependent genes. Affymetrix probe sets were mapped to Gene Ontology (GO) Biological Process and Molecular Function categories [DAVID, Ref [23]]. For each group, the top 5 ranked processes or functions are tabulated with the number of probe sets and the percentage of the dataset (%) that map to the given process or function, and the statistical significance for enrichment (p value).

**Early**				**Middle**			
**Molecular Function**	**Num**	**%**	**p Value**	**Molecular Function**	**Num**	**%**	**p Value**

CYTOKINE ACTIVITY	6	31.6	1.44E-06	PROTEIN BINDING	5.000	29.400	0.048
RECEPTOR BINDING	7	36.8	8.1E-06	SUGAR BINDING	2.000	11.800	0.092
CHEMOKINE ACTIVITY	4	21.1	1.37E-05	CARBOHYDRATE BINDING	2.000	11.800	0.096
CHEMOKINE RECEPTOR BINDING	4	21.1	1.37E-05				
G-PROTEIN-COUPLED RECEPTOR BINDING	4	21.1	1.52E-05				
							

**Late**				**Paradoxical**			

**Molecular Function**	**Num**	**%**	**p Value**	**Molecular Function**	**Num**	**%**	**p Value**

PEPTIDE TRANSPORTER ACTIVITY	2	12.5	0.005	CTD PHOSPHATASE ACTIVITY	2.000	12.500	0.044
PRIMARY ACTIVE TRANSPORTER ACTIVITY	3	18.8	0.059	Mg-DEPENDENT Ser/Thre PHOSPHATASE	2.000	12.500	0.044
PROTEIN BINDING	5	31.3	0.063	MYOSIN PHOSPHATASE ACTIVITY	2.000	12.500	0.044
				PROTEIN PHOSPHATASE TYPE 2B ACTIVITY	2.000	12.500	0.044
				PROTEIN PHOSPHATASE TYPE 2C ACTIVITY	2.000	12.500	0.044

**Table 2:** Functional classification of NF-κB-dependent genes.

**Function**	**Name**	**GenBank**	**Locus**	**Pr(F)**	**Cluster**	**Function**	**Name**	**GenBank**	**Locus**	**Pr(F)**	**Cluster**
**Anti-apoptosis**						**Receptors**	TAP1	X57522	6p21.3	1E-08	**Late**
	BID	AF042083	22q11.1	1E-07	**Middle**		TAPBP	AF029750	6p21.3	1.2E-07	**Late**
	BIRC2	U37547	11q22	0.00017	**Middle**		NK4	AA631972	16p13.3	4E-08	**Late**
	TNFAIP3	M59465	6q23	4.67E-11	**Early**		KCNG1	AL050404	20q13	1.21E-10	**Paradox**
**Cytokine**							ITGB5	X53002	3q21.2	0.003318	**Paradox**
	IL8	M28130	4q13	4.02E-08	**Early**		GPR49	AF062006	12q22	0.006668	**Paradox**
	IL6	X04430	7p21	8.89E-08	**Early**		CHRNB4	U48861	15q24	0.00637	**Paradox**
	TNF	X02910	6p21.3	0.002926	**Early**		F2RL1	U67058	5q13	2.67E-05	**Paradox**
	CXCL1/Gro-a	X54489	4q21	6.04E-07	**Early**		AQP3	N74607	9p13	0.008818	**Biphasic**
	CXCL3/Gro-g	M36821	4q21	4.62E-08	**Early**	**Signaling**					
	CXCL2/Gro-b	M36820	4q21	9.88E-15	**Early**		IkBe	U91616	6p21.1	1.10E-10	**Middle**
	CCL20/Exodus-1	U64197	2q33	1.96E-07	**Early**		BCL3	U05681	19q13.1	8.44E-05	**Middle**
**Growth Factors/Secreted Proteins**							TRAF2	U12597	9q34	0.001067	**Middle**
	TNFAIP2/B94	M92357	14q32	1.20E-09	**Middle**		TRAF1	U19261	9q33-q34	5.87E-07	**Late**
	Comp B	L15702	6p21.3	1.2E-08	**Middle**		TRAF3	U21092	14q32.33	0.000139	**Late**
	EFNA1	M57730	1q21	8.34E-05	**Early**		IkBa	M69043	14q13	5.55E-15	**Early**
	Follistatin	M19481	5q11.2	2.26E-05	**Paradox**		PTGS2	U04636	1q25.2	3.43E-08	**Early**
	CTGF	X78947	6q23.1	3.6E-06	**Paradox**		PPP1R3C	N36638	10q23-q24	0.000734	**Paradox**
	SCGF	AF020044	19q13.3	0.002939	**Paradox**		DUSP4	U48807	8p12	8.4E-06	**Paradox**
**Metabolic**							PTGES	AF010316	9q34.3	0.002286	**Late**
	SOD2	X07834	6q25.3	2.98E-07	**Middle**	**Transcription factor**					
	GCH1	U19523	14q22.1	3.93E-07	**Middle**		NF-kB1	M58603	4q24	1.73E-08	**Middle**
	GFPT2	AB016789	5q34-q35	8.00E-10	**Middle**		RELB	M83221	19q13.32	4.00E-14	**Middle**
	TIMP2	U44385	17q25	0.009703	**Paradox**		NFKB2	X61498	10q24	3.62E-14	**Late**
	HES1	L19314	3q28	0.000133	**Paradox**		REL	X75042	2p13	0.00011	**Early**
	CYB5	L39945	18q23	0.005638	**Biphasic**		IRF1	L05072	5q31.1	1.79E-06	**Early**
	PSMB9	AA808961	6p21.3	1E-09	**Biphasic**		TRIM16	AF096870	17p11.2	0.000221	**Late**
	PSMB8	X87344	6p21.3	0.007828	**Biphasic**	**Unknown**					
**Receptor/cell surface**							Unknown	HG371-HT26388	-	7.53E-05	**Late**
	KLRC3	AJ001685	12p13	0.000029	**Middle**		TNIP1/Naf-1	AJ011896	5q32	1.00E-11	**Late**
	SDC4	D79206	20q12	6.60E-08	**Middle**		PLAU	X02419	10q24	0.000399	**Early**
	SLC7A2	D29990	8p22	0.004933	**Middle**		OLFML2A	AL050002	9q34.11	0.000027	**Paradox**
	CD83	Z11697	6p23	1.50E-11	**Middle**	31	HBE1	AI349593	11p15.5	2E-07	**Paradox**
	IFNGR2	U05875	21q22.11	4.18E-06	**Middle**		chimeric	Y15915	-	0.005238	**Paradox**
	ECE1	Z35307	1p36.1	0.004074	**Middle**		DLX2	U51003	2q32	0.008043	**Paradox**
	KLRC2	AJ001684	12p13	0.000822	**Late**		Transgelin	D17409	11q23.2	0.00012	**Paradox**
	ICAM1	M24283	19p13.3	0.000124	**Late**		IFI35	U72882	17q21	0.004532	**Biphasic**
	IL27RA	AI263885	19p13.11	6E-09	**Late**		MVP	X79882	16p13.1	0.004212	**Biphasic**

The 74 NF-κB dependent probe sets were analyzed. Duplicate probe sets (e.g., those mapping to the same gene) were eliminated and unique genes tabulated. For each gene identified, the primary cellular function (Function), the common name (Name), the Genbank Accession number (GenBank), the chromosomal locus (Locus), the p-value indicating its significance that its expression is affected by NF-κB [Pr(F)], and its Cluster location (Cluster). Clusters are colored according to indicated expression pattern. Abbreviations used are: BID, BH3 domain interacting agonist; BIRC3, IAP homolog 3; TNFAIP3, TNF alpha induced protein3 (A20); IL, interleukin; CXCL, CXC motif ligand; CCL, CC motif ligand; Comp B, complement factor B; EFNA1, ephrin-A1: CTGF, connective tissue growth factor; SCGF, stem cell growth factor; SOD, superoxide dismutase; GCH1; GTP cyclohydrolase; GFPT2, glutamine-fructose-6-phosphate transaminase 2; PSMB, proteasome subunit; CYB5, cytochrome B5, HES, hairy enhancer of split; TIMP, tissue inhibitor of metalloprotein;, KLRC, SDC4, syndecan 4; SCLC7A2, human cationic transporter; IFNGR2, IFN gamma receptor 2; ECE, endothelial converting enzyme; KLRC2, natural lectin killer receptor 2; ICAM, intercellular adhesion molecule; IL27RA, interleukin 27 receptor alpha; TAP, transporter of antigen peptide; TAPBP, TAP binding protein; NK4, natural killer receptor 4; AQP3, aquaporin 3; KCNG1, potassium voltage-gated channel; ITGB5, integrin beta 5; GPR49, orphan G coupled receptor 49; CHRNB4 beta 4 nicotinic acetylcholine receptor; F2RL1, proteinase activated receptor -2; BCL-3, B cell lymphoma 3; TRAF, TNF receptor associated factor; PTGES, prostaglandin endoperoxide synthase; PTGS2, prostaglandin synthase 2; PPP1R3C, regulatory subunit of protein phosphatase 1; DUSP, dual specificity phosphatase; IRF, interferon response factor; TRIM 16, tripartite motif containing-16/estrogen responsive B-Box protein; TNIP/Naf-1, TNF inducible protein/Nef-associated factor-1; MVP, major vault protein; PLAU, urokinase plasminogen-activator gene; OLFML2A, olfactomedin-like 2A; HBE1, hemoglobin epsilon chain; DLX2, distal-less homeobox2.

**Figure 5:**
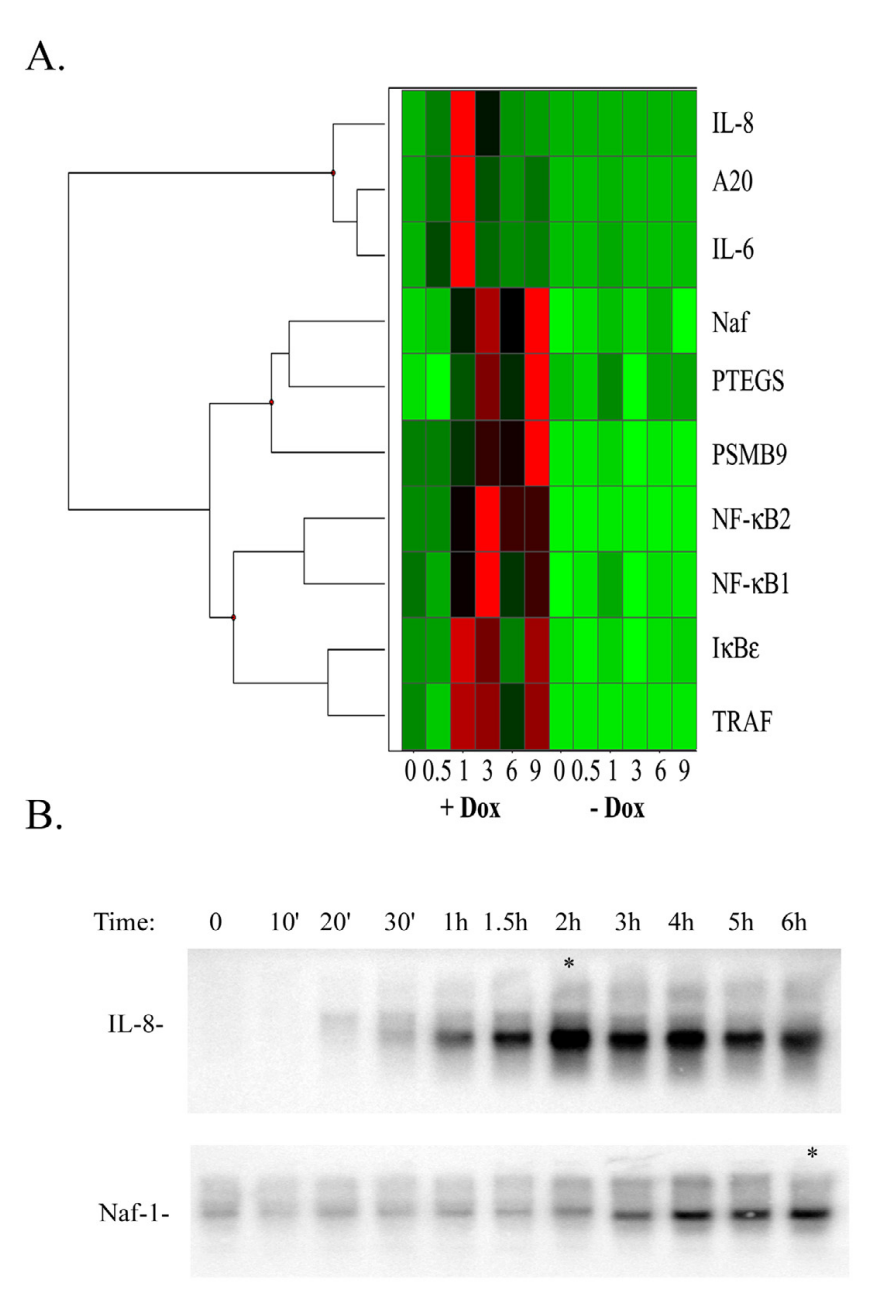
**(a) **IL-1 induces sequential cascades of NF-κB dependent gene expression. HeLa^tTA/FLAG-IκBα Mut ^cells were plated in parallel in the absence or presence of Dox (2 μg/ml) and stimulated with IL-1α. Changes in mRNA abundance (normalized by 18S) was then determined by Q-RT- PCR from total RNA. Shown is a Z-score representation, where red corresponds to Z > +2.5, green indicates Z < 0, and black indicates Z > 0.5. The common name of each gene is indicated at right. **(b) **TNF sequential cascades of NF-κB dependent gene expression in MRC-5 fibroblasts. Human MRC-5 fibroblasts were stimulated for the times indicated at top with TNFα (20 ng/ml) and RNA extracted. Shown is a northern blot hybridization of 20 μg RNA using probes specific to IL-8 (top) and Naf-1 (bottom). Asterix indicates apparent plateau of gene expression.

To determine whether the NF-κB-dependent gene expression cascades produced by TNFα are observed with other NF-κB activating stimuli, we stimulated HeLa^tTA/FLAG-IκBα Mut ^with IL-1α. IL-1α shares the ability to rapidly activate the IKK- NF-κB pathway with indistinguishable kinetics [20]. The mRNA expression profiles displayed as a heat map shows four expression profiles (Figure 5). IL-8, TNFAIP3/A20 and IL-6 (Early genes in response to TNF) were also rapidly induced by IL-1, peaking 1 h after stimulation. The genes encoding Naf-1, PTGES and PSMB9 (Late genes in TNF response) peaked 9 h after IL-1 stimulation. NF-κB1, and NF-κB2 constituted a Middle expression group. Together we conclude that similar temporal expression programs and NF-κB-dependence are seen in response to IL-1 signaling for members of the Early- and Late NF-κB-dependent genes. To partially address whether these expression profiles could be observed in other cell types, a time course experiment of TNFα – stimulated MRC-5 fibroblasts was analyzed for changes in a representative member of the Early gene group (IL-8) and a member of the Late gene group (Naf-1) by Northern blot analysis. As seen in Figure 5b, IL-8 is induced with an apparent plateau 2 h after TNF stimulation. Conversely, Naf-1 expression is not detectably induced at 2 h, but rather begins to increase after 3 h of stimulation, apparently reaching a plateau 6 h and later after stimulation. These findings suggest that these waves of genomic NF-κB responses can be observed in other cell types.

**Figure 6:**
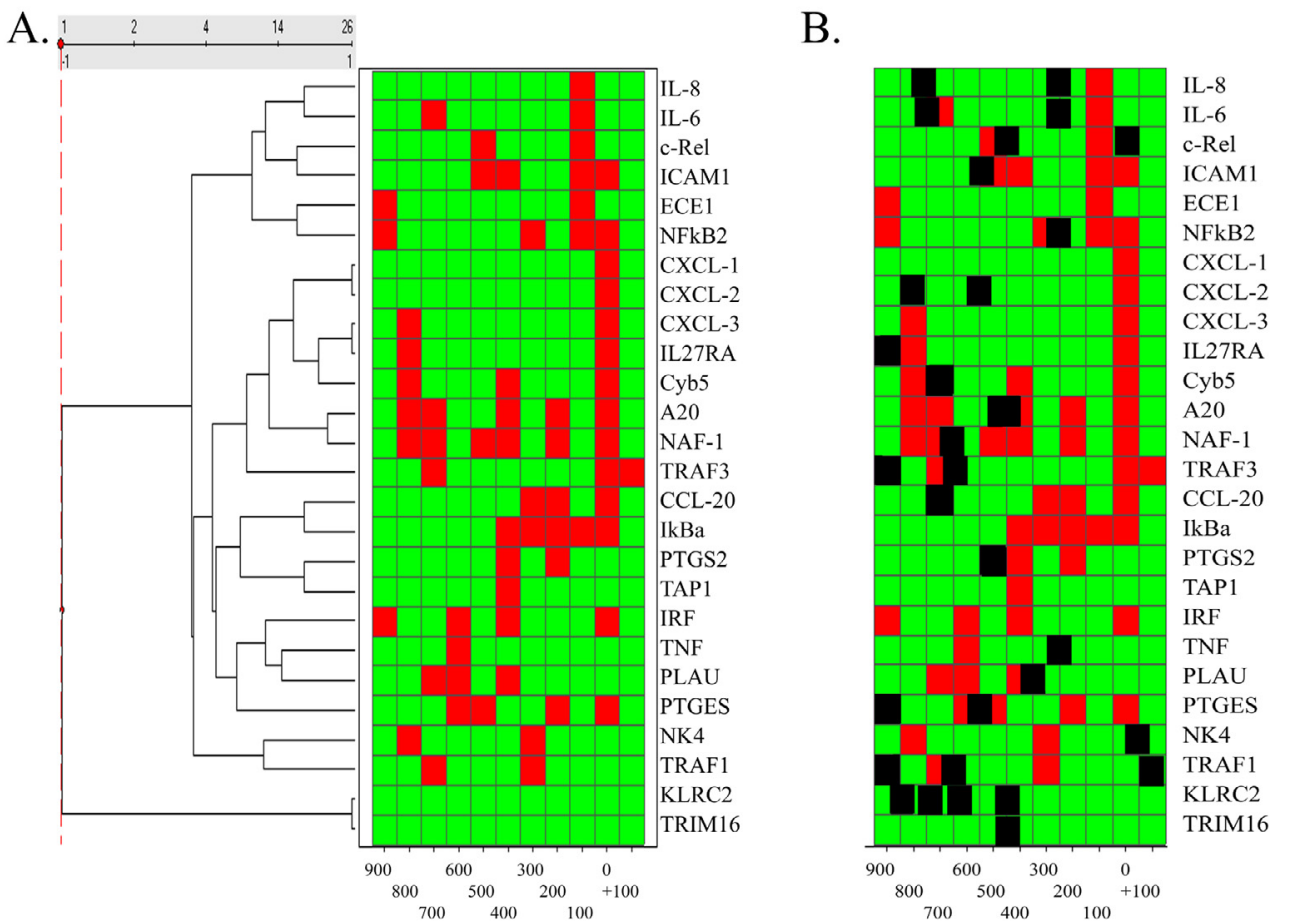
**(a) **Hierarchical clustering of high-affinity NF-κB DNA-binding sites. The probability over 100 bp intervals for finding a high-affinity NF-κB-binding site was used for hierarchical clustering (data from Table I) of the early and late NF-κB dependent gene promoters. Data is shown as a heat map, where green = 0, red = 1. The common name of each gene is shown at right. Note that there is no separation of early and late gene promoters based on the pattern or location of the NF-κB-binding sites. **(b) **Co-occurrence of high-affinity NF-κB- and AP-1 DNA-binding sites. Superimposed on the NF-κB binding site analysis is the presence and location of high-affinity AP-1 DNA-binding sites. The location of each AP-1 DNA-binding site is indicated in black.

The promoters of the Early and Late response groups were subjected to bioinformatics analysis, to determine whether the kinetics of NF-κB-inducible transcription was a function of the location or number of high-affinity NF-κB-binding sites [24]. Within 1000 bp of the transcription start site, between 1 to 6 high-affinity NF-κB-binding sites were found in both expression groups [see Additional files 2, 3]; when subjected to unsupervised hierarchical clustering, neither the location or number of NF-κB-binding sites was apparently predictive of target gene expression pattern (Figure 6a). For example, Naf-1, a Late gene, co-clustered with A20, an Early gene (Figure 6a). Both promoters had >5 putative high-affinity NF-κB DNA-binding sites in their promoters. To initially address the possibility that the combinatorial context in which the NF-κB site was located may determine its pattern of response, we examined whether AP-1 binding sites were enriched in the Early gene promoters. For example, previous promoter analyses from our lab have shown that the presence of AP-1 binding site affects the magnitude of IL-8 gene induction in response to TNFα [25]. The location of putative high-affinity AP-1 binding sites in relationship to the NF-κB binding sites are shown in Figure 6b. Although AP-1 binding sites are frequent in the promoters analyzed, with 31 sites found in the 26 promoters analyzed, the frequency of those containing AP-1 was not different between the expression groups. Here, 10 of 13 Early gene promoters contained at least one AP-1 site, and exactly the same number of Late gene promoters (10/13) contained them.

**Figure 7:**
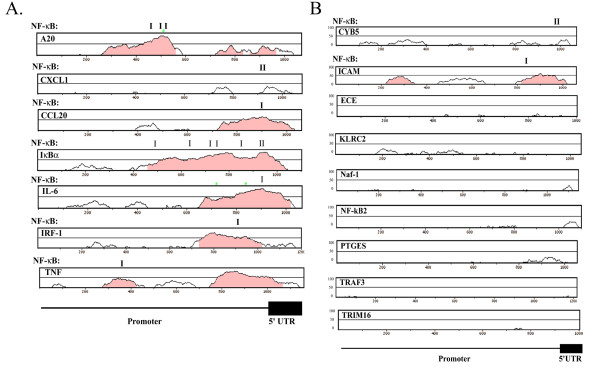
Phylogenetic analysis of NF-κB dependent promoters. **(a) **Early gene promoters. Promoters spanning from -1000 bp to the first nontranslated exon were aligned between human and mouse genes. Shown are the VISA identity curves [49]. For each curve, the percent sequence conservation is plotted over a sliding 20 base pair window (from 0–100% identity). Shaded regions indicated significant regions of sequence conservation. The location of NF-κB-binding sites within these conserved domains are displayed at top (location indicated by I). The presence of AP-1 sites is indicated by green asterix (*). **(b) **Late gene promoters. For each late gene promoter indicated, analysis as in 7a.

However, when the location of NF-κB-binding sites located within phylogenetically conserved domains was considered, striking differences between the two groups emerged. For the Early genes, promoter alignments between human and mouse genes showed that the NF-κB-binding sites were highly conserved, where the A10, CXCL-1, CCL-20, IκBα, IL-6, IRF-1 and TNF genes contained NF-κB-binding sites, representing 7 of the 9 genes amenable for analysis (Figure 7a). Conversely, for the Late genes, only CYB5 and ICAM-1 had NF-κB-binding sites within phylogenetically conserved domains, representing only 2 of the 9 genes (Figure 7b). Together, these data indicate that the Early gene promoters may be under selective conservation pressure to contain NF-κB-binding sites, whereas the Late gene promoters may not be. To further explore the question of co-occurrence of AP-1-binding sites, the frequency of AP-1 sites in phylogenetically conserved domains was also examined. As seen by the green asterixes in Figure 7a, only two phylogenetically conserved domains in Early genes contained high-affinity AP-1-binding sites. More work will be required to understand the biological significance of these apparent differences in binding patterns between the two groups.

**Figure 8:**
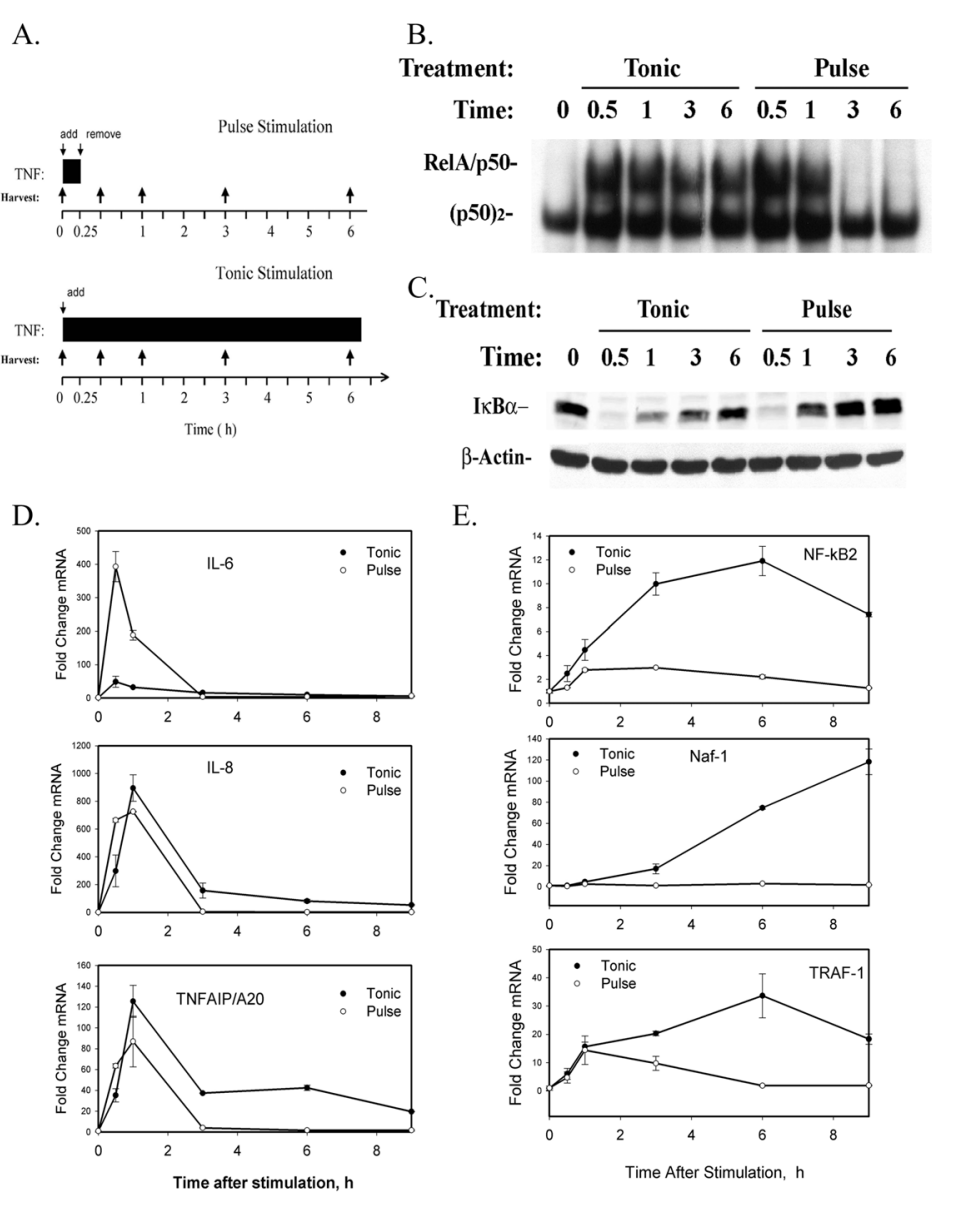
Late gene expression requires the NF-κB oscillatory mode. **(a) **Experimental Strategy. Schematic diagram of the tonic and pulse stimulation paradigm. Parallel plates of cells were stimulated with TNF continuously ("tonic" treatment), without removing the agonist. Pulse stimulated cells were exposed to TNF to activate the NF-κB pathway (activation is maximal within 15 min of stimulation), whereupon the agonist is removed from the medium. At identical times after application of the stimulus, cells are harvested for gel shift (Figure 8b) or Q-RT-PCR (Figures 8c, d). **(b) **NF-κB-binding in tonic- vs pulse-stimulated cells. Nuclear extracts from tonic- or pulse stimulated HeLa cells were prepared and NF-κB-binding measured. Shown is an autoradiogram of the bound NF-κB complexes by EMSA. The specific NF-κB/Rel A and NF-κB1 complexes previously identified by supershift analyses are indicated at left (see Ref [21] for further details). **(c) **IκB proteolysis and resynthesis in tonic- vs pulse-stimulated cells. Cytoplasmic extracts from tonic- or pulse stimulated HeLa cells were prepared and abundance of IκB determined by Western blot. IκB is rapidly proteolyzed, with both treatments, however, the steady state levels are reduced 3 and 6 h in tonic treated cells compared to those pulse-treated. **(d) **Early gene expression profiles. HeLa cells were treated as in Figure 8a, total RNA extracted and mRNA abundance (normalized by 18S) determined by Q-RT- PCR. For each of the indicated mRNA transcripts, values are expressed as fold change relative to unstimulated cells and plotted on a logarithmic scale. **(e) **Late gene expression profiles. Samples obtained as in Figure 8d. The mRNA transcript measured is indicated for each plot.

TNF can induce two distinct modes of NF-κB activation patterns- a single, synchronized "monophasic" NF-κB translocation *vs *a series of damped, desynchronized oscillations ("oscillatory") whose differential effects on cellular genetic response has not been explored [11,14]. Pulse TNF stimulation rapidly activates IKK briefly over 5–15 min, after which the kinase inactivates, thereby allowing newly resynthesized IκBα to recapture activated NF-κB and return it to its inactivated cytoplasmic form. Conversely, tonic TNF stimulation produces a low level of persistent IKK activity. This persistent IKK activity produces continuous IκBα proteolysis and NF-κB binding [14]. To illustrate, the DNA binding profiles of "pulse" TNFα stimulation (15 min, Figure 8a) were then compared with "tonic" TNFα stimulation. Over the first 1 h, NF-κB DNA binding activity in EMSA was indistinguishable between the "tonic" and "pulse" TNF stimulation (Figure 8b). However, after 3 h, NF-κB activated by pulse TNF stimulation is no longer detectable in the nucleus, being relocated into the cytoplasm, whereas tonic TNF stimulation produced a low level of NF-κB binding (Figure 8b; compare 3- and 6 h Tonic vs Pulse stimulated cells). Although high resolution single-cell fluorescence microscopy indicates this is due to a series of dampened oscillations, the oscillations have desynchronous cycle times and presents as an apparently tonic binding pattern in the homogenated cell population (Figure 8b). As expected, cytoplasmic IκBα is rapidly reduced within 30 min in cells subjected to either pulse or tonic stimulation, but only those subjected to tonic treatment show persistent IκB proteolysis (see Western blot in Figure 8c), producing an oscillatory NF-κB translocation profile (compare with Figure 8b). Using these two stimulation modes, we tested their effect on the Early and Late gene expression profiles by Q-RT-PCR. For the Early genes, we found that the expression patterns for IL-8 and TNFAIP3/A20 gene expression were quite similar (Figure 8d). Surprisingly, IL-6 response to pulse stimulation was much greater than that of identically cultured cells that were tonically TNF stimulated (Figure 8d). Cells in the pulse-treated plates are washed in PBS to remove the TNFα ligand after the 15 min exposure time. It may be possible that a secreted TNFα-inducible inhibitor of IL-6 expression (such as an arachidonic acid metabolite) is removed during this processing, accounting for the enhanced IL-6 expression. Nevertheless, and in marked contrast, Late gene expression patterns were significantly reduced in response to pulse stimulation. Tonic TNF stimulation produced a 12-fold induction of NF-κB2 and 120-fold induction of Naf-1 mRNAs, whereas the pulse stimulation produced less than 2-fold mRNA induction for either gene (Figure 8e). Also, the Early expression of TRAF-1 (15 fold at 1 h) was similar for both treatment conditions; however at later times, TRAF-1 expression returned to unstimulated values. Together these data indicate that expression of the Late genes are dependent on tonic stimulation producing continuous oscillatory NF-κB activity, and suggest that the Late genes are recruited into activated expression modes by time-dependent NF-κB exposure.

## Discussion

TNFα is a potent inflammatory and immunomodulatory cytokine expressed by macrophages, monocytes, neutrophils, T-cells and natural killer (NK)-cells following stimulation by bacterial endotoxin. Upon binding to high-affinity cell surface receptors, TNFα activates the expression of secondary cytokine cascades and adhesion molecules that, in turn, play important roles in tissue inflammation by coordinating leukocyte activation, chemotaxis and cell death [1,2,26,27]. The intracellular signaling pathways in response to TNF are well understood. Ligation of TNFRI induces protein recruitment to its cytoplasmic death domains, assembling a submembranous signaling complex composed of TRADD, FADD, TRAF2 and other proteins. These, in turn, activate two divergent intracellular signals, the JNK-AP-1 and the IKK-NF-κB pathways responsible for producing homeostatic genomic responses. Although the IKK-NF-κB pathway is critical for inducing tissue inflammation and preventing TNF-induced programmed cell death, surprisingly little is known about its downstream gene targets and their kinetics of induction. In this study, we have systematically analyzed the kinetics of NF-κB-dependent gene expression. Our findings suggest that NF-κB controls distinct groups of target genes whose pattern of expression appear to be an orchestrated cascade of Early, Middle and Late target gene responses. These kinetically separable waves of NF-κB-dependent gene expression control distinct biochemical processes, with the Early gene group primarily encoding for cytokines that mediate TNF's ability to amplify local cytokine cascades in inflamed tissue. Moreover, we find the orchestration of distinct temporal gene expression cascades is a general feature of cytokine-induced NF-κB activation, being also observed in response to stimulation with IL-1. Undoubtedly cell type-specific influences may affect the precise timing of expression and composition of the kinetic groups that we have identified here for epithelial cells, we nevertheless find similar distinct temporal profiles of representative member of the Early and Late gene groups in unrelated human MRC-5 fibroblasts. Finally, our study identifies differences in gene expression depending on NF-κB activation modes that affect target genes within non-phylogenetically conserved regulatory domains. These findings shed important new insights into the genetic responses to cytokine action.

The Early genes are enriched in cytokines and regulatory components of the IKK-NF-κB pathway as analyzed by Gene Ontology, Ingenuity pathway analysis, and expert classification. An important biological property of TNF is to initiate the cytokine cascade in target cells, where the expression of secondary (downstream) cytokines are produced, each with their own distinct biological properties [1,27]. In this manner, TNF amplifies the inflammatory process. We find that a major part of the Early gene group is the CXC chemokine family. CXC chemokines are the numerically largest of the chemokine families, responsible for inducing migration of neutrophilic leukocytes, stimulating wound healing, initiating angiogenesis and promoting tumorigenesis [28]. In addition, a CC chemokine, CCL-20, is responsible for stimulating monocytes and dendritic cells [29]. Another Early gene, the cytokine IL-6, induces B cell differentiation and is a major mediator of the hepatic acute phase reaction. Therefore, TNF stimulation of epithelial cells rapidly induces secondary cytokine cascades that control leukocyte trafficking, wound healing, angiogenesis, and systemic inflammation. Our phylogenetic analysis shows that the Early gene promoters contain NF-κB-binding sites in evolutionarily conserved regions between human and mouse, perhaps suggesting existence of selection pressure for this rapid TNF response. Moreover, since chemokine activity is produced as a major portion for the most rapidly expressed genes, the primary responses of the TNF-stimulated epithelium appear to be the paracrine propogation of the inflammatory response, with the induction of homeostatic factors a secondary priority for the cell.

The other important members of the Early genes encode intracellular regulatory molecules involved in inhibition of the IKK-NF-κB pathway itself. The NF-κB pathway is tightly controlled by negative feedback loops at multiple steps in its signaling pathway [14,30]. One level of feedback inhibition involves the inactivation of nuclear NF-κB and return to its cytoplasmic localization, a process termed the NF-κB-IκB autoregulatory loop [13,15]. In this loop, activated NF-κB produces enhanced expression of IκBα mRNA. IκBα protein is then replenished to bind and inactivate NF-κB/Rel A, returning it back into to the cytoplasm to restore homeostasis. At a second level, activated NF-κB induces inhibitors of the activated IKK complex. This inhibition is mediated by the TNFAIP3/A20 protein, a ubiquitin ligase that associates with RIP and mediates its proteasomal degradation [31], resulting in inhibition of IKK signal [14,31-34]]. Together, these observations indicate that an additional effect of the Early NF-κB response is to terminate the TNFR-IKK-NF-κB signaling pathway at several levels whose effect is to restore cellular homeostasis.

Conversely, the Late gene group encodes adhesion molecules (ICAM, KLRC2), MHC I antigen processing/presentation (TAP, TAPBP). These molecules play important roles in cytotoxic T cell mediated cytolysis. The finding that tonic TNF stimulation is required for adhesion molecule expression and MHC I antigen presentation suggests that tonically TNF stimulated cells, such as those produced in the context of persistent infection, would be targeted for enhanced immune recognition and clearance. Also in this group, the TRAF signal adapter molecules couple TNF receptors to intracellular responses. TRAF1 is distinct from other TRAF isoforms in that it apparently serves to protect cells from apoptosis and plays a role in the negative feedback regulation of receptor signaling [35]. Similarly TRAF3 has inhibitory functions to those of TRAF 2/6 in TNF induced NF-κB activation [35]. In this regard, TRAF-1 and 3 dependence on tonic TNF stimulation suggests a mechanism how the cell attempts to restore homeostasis in the presence of a strong pro-apoptotic stimulus by additional down- regulation of the TNFR-IKK signaling pathway. Our new findings that Late gene expression is dependent on tonic TNF stimulation is mechanistically significant because it means that TNF may produce distinct phenotypic responses depending on the stimulus duration.

Although the mechanisms underlying the different patterns of Early and Late gene expression control were not the focus of this study, several findings merits further discussion. Our preliminary analyses indicate that expression of both classes of genes is absolutely dependent on NF-κB translocation, because expression of both groups is completely blocked by overexpression of the nondegradable IκBα inhibitor (Figures 2, 3). Bioinformatic analysis shows that both groups contain high-affinity NF-κB-binding sites (Figures 6, 7), many of which have been experimentally verified [21,36]. Moreover, we show here that members from both groups inducibly bind NF-κB within their native chromatin environment (Figures 3c,d). Additionally, we previously showed that TNFα robustly induces expression of three members of the Middle gene group (IκBε, NF-κB1, and RelB), and three members of the Late gene group (TRAF-1, NF-κB2, and Naf-1) in the absence of new protein synthesis [21]. Protein synthesis independence excludes paracrine factors mediating Late gene expression in epithelial cells, unlike those seen in other cell types [37]. Finally, we have previously shown that expression of a constitutively active NF-κB/RelA transactivator is sufficient to activate expression of representative Middle and Late genes, excluding a requirement for other TNF-induced signaling pathways in expression of these genes. Together, these data strongly argue that TNFα – induced NF-κB binding to high-affinity DNA-binding sites in the Late gene promoters is necessary and sufficient for their expression. NF-κB, therefore, is a direct regulator of Late gene expression.

Recent studies suggesting that NF-κB binding occurs in two distinct "waves" in LPS-stimulated macrophages [38] raises the question whether Late gene expression could be due to different rates of NF-κB recruitment. Unfortunately, our findings do not support this as a mechanism controlling Late gene activation by NF-κB in epithelial cells. For example, we have previously shown that the kinetics of the potent transactivating NF-κB/RelA subunit binding to the Late gene, Naf-1, is rapid and indistinguishable from that for the Early gene, IκBα [21]. It is still possible that other Late genes not yet tested are bound by RelA more slowly, but at least we can conclude that differences in rate of NF-κB binding cannot account for the late pattern of Naf-1 expression. Alternatively, expression differences could be due to different compositions of NF-κB subunit binding to the Early and Late gene promoters. Although the ChIP assays presented in Figures 3c and 3d show that the RelA, c-Rel and NF-κB1 subunits bind similarly to the Early and Late genes, NF-κB2 appeared to be binding more strongly to the late genes. It therefore is possible that exchange of various transactivating subunits for NF-κB2 may occur later in the time series, a possibility that will require further investigation.

Another possible explanation for the different rates of promoter activation may be through the environment in which the NF-κB binding sites are located in the Early and Late gene promoters. The rate of response of some NF-κB dependent genes has been suggested to be modified by adjacent transcription factor regulatory sites. In our previous studies of IL-8 gene expression, we found that the magnitude of its TNF-induced transcriptional response is partly dependent on an intact upstream AP-1 binding site [25]. Although our preliminary bioinformatic analysis does not indicate any differences in the frequency of co-regulatory AP-1 binding sites between the two groups (Figure 6b), other sites or combinations of sites may be important. For example, a rapid transcriptional response of the A20 gene has been suggested to be due to a "pre-assembled" pre-initiation complex that is nucleated by the SP-1 transcription factor [39]. In this way, the A20 promoter is poised for rapid transcriptional induction when NF-κB is activated. It will be interesting to compare whether the Early genes are pre-loaded with TFIID or RNA Polymerase by ChIP, and whether these patterns are different from the Late gene promoters. However, we note from our previous genomic footprinting studies have shown that TNF induces both NF-κB- and TFIID binding simultaneously to the IL-8 promoter in epithelial cells [18]. We therefore think SP-1 mediated promoter pre-loading is not likely to be a universal mechanism explaining Early gene expression.

Another possible explanation for the delay in Late gene expression is that this group undergoes an additional rate-limiting step necessary for promoter activation after NF-κB binding has occurred. This step is apparently dependent on a TNF stimulation protocol that induces oscillatory NF-κB binding behavior. In a previous mathematical treatment of this phenomenon at a single cell level, we demonstrated that the Late gene expression profiles can be simulated using a theoretical construct of two sequential activator binding steps, the first one being NF-κB [40]. This second activator, yet to be experimentally identified, could be chromatin modification, nucleosomal re-positioning, pre-initiation complex formation, or coactivator recruitment (reviewed in [41-43]]). These possibilities will require further experimentation.

Another conclusion from our study is that Early gene expression is being actively terminated. Comparing the microarray and Q-RT-PCR profiles with NF-κB binding profiles show that IL-8 expression is falling to control levels 3 h after TNF stimulation, even though NF-κB binding continues to be detectable at these times. Both EMSA (Figure 8) and ChIP assays show NF-kB binding is strongly at these times (See Figure 7F in [21]). To our interpretation, these findings indicate a repressive activity is being recruited to the Early gene promoters during the evolution of the TNF response, an activity or factor which has yet to be experimentally identified [see also discussion in [40]].

Finally, we have not addressed regulation of the Paradoxical genes. These genes are not affected by TNF stimulation in the presence of intact NF-κB signaling pathway (Figure 2), but are induced by its absence. One possibility is that they represent a group of genes whose expression is tonically inhibited by basal NF-κB nucleo-cytoplasmic shuttling. This could be through a competition for rate-limiting, shared, coactivators. In the absence of NF-κB translocation, these limiting coactivators are now able to bind and activate expression of the Paradoxical genes.

## Conclusion

This study is the first systematic dissection of the NF-κB response profiles downstream of TNF. We have found evidence for temporal waves of NF-κB-dependent target expression encoding distinct molecular functions. The expression profiles are stimulus-independent, being induced in the same coordinated cascades in response to IL-1. Finally, we have identified a subnetwork of the NF-κB response program whose expression is dependent on its oscillatory mode of activation. This finding is significant in that it indicates distinct cellular phenotypes can be produced depending on the duration of TNF stimulation.

## Methods

### Cell culture and treatment

HeLa^tTA/FLAG-IκBα Mut^, Tet-transactivator (tTA)-expressing HeLa cells stably transfected with a Tet Operator controlled non-degradable IκBα (IκBα Ser^32^Ala/Ser^36^Ala) plasmid, were cultured as described [22]. For pulse TNF stimulation, cells stimulated with 25 ng/ml recombinant TNFα for 15 min, and rapidly washed 3 times with PBS before returning to culture medium. For tonic stimulation, 25 ng/ml TNF was added to the culture medium and left for indicated times prior to harvest.

### RNA analysis

Twenty micrograms acid guanidium-phenol extracted RNA was analyzed by Northern blot as previously described [21]. The washed membrane was exposed to a Molecular Dynamics PhosphorImager cassette for quantitation. Quantitative real-time reverse transcriptase-polymerase chain reaction (Q- RT-PCR) assays used commercially available primer and probe sequences (ABI, P/N 4331182). For TNFAIP3/A20, the probe sequence was 5'-CAATTGCCG TCACCGTTC-3'; the forward primer was 5'-AGCTTGTGGC GCTGAAAAC-3', and reverse primer was 5'-ACTGAGAAGTG GCATGCATGAG-3'. The cycling parameters for one-step RT-PCR were: reverse transcription 48°C for 30 min, AmpliTaq activation 95°C for 10 min, denaturation 95°C for 15 s, and annealing/extension 60°C for 1 min (repeat 40 times) on an ABI7000 thermocycler. Duplicate CT values were analysed using comparative CT(ΔΔ CT) method. The amount of target (2 ^-ΔΔCT^) was obtained by normalizing to an endogenous reference (18S RNA) and relative to a calibrator (one experimental sample).

### Oligonucleotide array data analysis

Four independent Hu95Av2 GeneChip (Affymetrix Inc, Santa Clara, CA) hybrdiziations were performed using RNA isolated from control (0 h), 1, 3 and 6 h TNF stimulated HeLa^tTA/FLAG-IκBα Mut ^cells in the presence or absence of Doxycyline (2 μg/ml). For comparison of the fluorescent intensity (Signal Intensity) values among multiple experiments, the Signal Intensity (SI) values for each "experimental" GeneChip were scaled to that of the "base" GeneChip and subjected to a 2 way analysis of variance with replications (ANOVA, Splus 6, Insightful Inc.). As seen in Figure 1 (supplementary information), 343 probe sets were changed by Dox treatment a p value [Pr(F)] of < 0.01. The probe sets were then filtered to identify any that showed a 3-fold difference in SI at any time during the TNF treatment (SI with NF-κB *vs *SI without NF-κB), identifying 74 probe sets being under NF-κB control. Agglomerative hierarchical clustering was performed using the Weighted Pair-Group Method with Arithmetic mean (WPGMA, Spotfire Array Explorer, v. 8, Spotfire Inc., Cambridge MA) using Euclidian Distance. The primary data has been deposited with GEO or can be found at our website [44].

Functional annotation mapping was performed using the NIAID DAVID database [23,45]). Pathways Analysis was performed using individual clusters as input into the Ingenuity Knowledge Base database [46]. NF-κB-dependent human promoters were obtained from the Human Genome Browser gateway using the Human May 2004 (hg17) assembly (UCSC Genome Bioinformatics Site, [47]. NF-κB-binding sequences were identified by TRANSFAC 4.2 filtering matrix scores by minimizing the sum the false positive and negative error rates. Human and mouse promoters were aligned using the VISTA genome browser 2.0 [48,49].

### Protein extraction and analysis

Nuclear and cytoplasmic proteins were fractionated as previously described [21]. 15 μg of nuclear extracts (NE) were subjected to Electrophoretic Mobility Shift Assay (EMSA) using the high-affinity NF-κB-binding site [22]. The complexes were fractionated on 6 % native polyacrylamide gels, dried, and exposed to Kodak X-AR film at 70°C. For Western blot, equal amounts of cytoplasmic protein were fractionated by SDS-PAGE and transferred to PVDF membrane. The membranes were incubated with affinity purified rabbit polyclonal antibodies to IκBα (Santa Cruz Biotechnology). Washed membranes were then incubated with IRDye 800 labeled anti-rabbit IgG antibodies (Rockland Immunochemicals, Gilbertsville, PA), and immune complexes quantified using the Odyssey Infrared Imaging system (LICOR Biosciences, Lincoln, NE.).

### Chromatin immunoprecipitation (ChIP) assay

The ChIP assay was as described [21]. On the day prior to experiment, 2–4 × 10^6 ^cells were plated in 0.5 % BSA containing growth medium. Cells were stimulated for indicated times, and sequentially crosslinked with disuccinimidyl glutarate and 1 % formaldehyde in serum-free medium for 15 min at 37°C. The cells were washed, transferred to Eppendorf tubes, and solubilized in 400 ml of SDS Lysis Buffer (1% SDS 10 mM Tris, ph 8.0, 1 mM EDTA) with protease inhibitor cocktail (Sigma Aldrich). The samples were sonicated 3 times, 15 sec at setting 2 until DNA fragments were 300–400 bp or less. Equal amounts of DNA were immunoprecipitated overnight at 4°C in ChIP dilution buffer (50 mM NaCl, 1 mM HEPES, pH 7.4, 1% IGEPAL-630, 10 % glycerol, 1 mM DTT) with 20 μg of indicated NF-κB subunit specific antibody (Santa Cruz Biotech) or IgG as indicated. Immunoprecipitates were collected with protein-A magnetic beads (Dynal, Inc), and washed sequentially with ChIP dilution buffer, high salt buffer, LiCl buffer and TE buffer (10 mM Tris, ph 8.0, 1 mM EDTA). DNA was eluted in 1 ml of Elution Buffer 1 % SDS in 0.1 M NaHCO_3_). Samples were de-crosslinked in 200 mM NaCl at 65°C, 1 h. DNA was phenol extracted, ethanol precipitated and used for PCR. PCR primers and conditions for semiquantiative PCR are in [21]. PCR products were fractionated by agarose gel chromatography and stained with ethidium bromide.

## Additional data files

Additional data are available with the online version of this manuscript. File 1 is the data showing the Z-Test analysis of TNF-regulated genes. Files 2 and 3 are the NF-κB-binding site predictions and human-mouse promoter mapping for the Early genes and Late genes, respectively.

## Supplementary Material

Additional File 1 Contains the table showing the Z-Test analysis of TNF-regulated genes. Click here for file

Additional File 2 Contains the NF-κB-binding site predictions and human-mouse promoter mapping for the Early genes. Click here for file

Additional File 3 Contains the NF-κB-binding site predictions and human-mouse promoter mapping for the Late genes. Click here for file
