# Supplementary material for: A TNF-induced gene expression program under oscillatory NF-κB control
Source: BMC Genomics. 2005 Sep 28;6:137. doi: 10.1186/1471-2164-6-137 (PMC1262712; doi:10.1186/1471-2164-6-137)
Supplement: Additional File 1 — Contains the table showing the Z-Test analysis of TNF-regulated genes. [file 1471-2164-6-137-S1.pdf]

Table 1. Z-Test analysis of TNF regulated genes.

		Middle			Late		
	**Time**	1 h	3 h	6 h	1 h	3 h	6 h
**Early**	1 h	< 1E -11			< 1E -11		
	3 h		< 1E -11			2.4E-09	
	6 h			8E-09			< 1E -11
**Middle**	1 h				4E-06		
	3 h					6.7E-16	
	6 h						4.4E-06

Table 1. Z-Test analysis of TNF regulated genes. The Signal Intensity Profiles of the The mean and variances for the normalized Signal Intensity measurements for Early Middle and Late genes were calculated and analyzed for differences in population means at each time point using two-tailed Z-Test. Shown are the P values for each comparison. For example, the population mean of Early gene group at 1 h is significantly different from that of the population mean of the Middle gene group at 1 h with a Z score probability of <1 E-11.
