# Supplementary material for: A TNF-induced gene expression program under oscillatory NF-κB control
Source: BMC Genomics. 2005 Sep 28;6:137. doi: 10.1186/1471-2164-6-137 (PMC1262712; doi:10.1186/1471-2164-6-137)
Supplement: Additional File 2 — Contains the NF-κB-binding site predictions and human-mouse promoter mapping for the Early genes. [file 1471-2164-6-137-S2.pdf]

Table 2. Early gene NF-κB prediction and human-mouse promoter mapping.

Table II: E		Human				Mouse				
**Promoter**	**Chr.**	**Location (bp)**	**NF-kB**	**MM**	**Sequence**	**Chr.**	**Location (bp)**	**NF-kB**	**MM**	**Sequence**
**A20**	6	138229274-138234057	161 (-)	0.828	cgggtgTCCCCtggga	10	18710180-18714780	542 (+)	0.803	ggcttGGAGAaccctg
			260 (-)	0.851	GAAATgcccg			813 (+)	0.807	gcttcCGAAAtgccca
			536 (+)	0.828	cacgcGGGGAcacccc			836 (+)	0.828	cacgcGGGGAcacccc
			747 (-)	0.966	ttggaaaGTCCCgt			943 (-)	0.96	GGAATttcca
			761 (-)	1	GGAAAtcccc			955 (-)	1	GGAAAtcccc
			1017 (-)	0.868	tgggacTTTGCgaaag			1000 (-)	0.949	GGAACtgccc
**CCL-20**	2	228503075-228504132	633 (-)	0.851	GGAATgttcc	1	83641204-83642245	57 (+)	0.878	ggtgtTTTCC
			780 (-)	0.85	caggatTCTCCccttc			900 (+)	0.954	atgggGGAAAaccccg
			905 (+)	0.954	aatggGGAAAacccca					
**CXCL-1**	4	75099144-75100222	922 (-)	1	cgggaaTTTCCctggc	5	89705281-89706230	246 (+)	0.977	tggacTTTCC
			948 (+)	0.99	cgggcTTTCC			328 (+)	0.84	ctttcGGGAAgttccc
								847 (-)	0.981	GGAAAcaccc
								865 (+)	1	gggaaTTTCC
								888 (-)	0.806	ctggagTTTCGagcat
**CXCL-2**	4	75329873-75330927	911 (-)	1	cgggaaTTTCCctggc	" "	" "			
			936 (+)	0.99	cgggcTTTCC					
**CXCL-3**	4	75269364-75270440	190 (-)	0.946	GGAAGttcca	" "	" "			
			920 (-)	1	cgggaaTTTCCctggc					
			946 (+)	0.99	cgggcTTTCC					
**IL-8**	4	74970371-74971410	859 (+)	0.978	tggaaTTTCC	" "	" "			
**IL-6**	7	22539060-22540121	241 (+)	0.86	ggaggATTCC	5	28412048-28413078	909 (-)	0.998	tgggatTTTCCcatga
			926 (-)	0.998	tgggatTTTCCcatga					
**TNF**	6	31650341-31651497	379 (-)	0.865	cggggcTGTCCcaggc	17	33697337-33698492	128 (+)	0.818	ggggaGGGGAatcctt
								250 (-)	0.866	GGAAAactca
								330 (+)	0.852	cgtgaATTCC
								361 (-)	0.842	tggggcTGCCCcatac
								478 (+)	1	ggggcTTTCC
								777 (+)	0.947	aggagATTCC

TABLE II, contd

IkBa	14	34943602-34944703	556 (+)	0.802	aaaacGGAAAggaccg	12	50679716-50680769	304 (-)	0.868	aggggtTCTCCaagct
			574 (+)	0.894	aggttGGCAAacccca			554 (+)	0.802	aaaccGGAAAagacct
			661 (-)	0.948	gggaaACCCC			656 (+)	0.927	ccggtGGGAAacccca
			724 (-)	0.942	GGAAGtaccg			723 (-)	0.927	GGAAGtacct
			755 (+)	0.965	aggacTTTCC			755 (+)	0.965	aggacTTTCC
			820 (-)	0.874	agggagTTTCTccgat			820 (-)	0.874	agggagTTTCTccgat
			915 (-)	0.997	ttggaaaTTCCCcg			905 (-)	1	GGAAAttccc
			942 (+)	0.831	cccagAGAAAtcccca			930 (+)	0.831	ccctgAGAAAtcccct
IRF	4	131853070-131855326	68 (+)	0.895	gccagGGGGAgtccca	11	53408817-53411027	257 (-)	0.866	GGAAAgcaca
			302 (+)	0.988	tgggaTTTCC			318 (-)	0.852	aaggtgTTCCCccatc
			400 (-)	0.819	agggggTCACCcctaa			419 (+)	0.956	tgggaGTTCC
			536 (+)	0.819	gcccgGGCGAtcccct			632 (+)	0.857	agaagGGGAAgaccat
			913 (+)	0.925	GGGGAatccc			924 (+)	0.931	GGGGAatccc
PTGS2	1	183381080-183382213	552 (+)	0.957	aggggATTCC	1	150029778-150030877	727 (+)	0.921	cagctGGAAAttccgt
			777 (-)	0.881	ggggacTACCCcctct					
c-Rel	2	61019451-61020626	415 (-)	0.926	GGAACcacct	11	23666257-23667537	478 (+)	0.935	tggcgCTTCC
			859 (+)	0.999	ccgcgGGAAAttcccc			565 (-)	0.941	GGAACcaccg
								714 (-)	0.831	gggggaTTTCTcaggg
								911 (-)	1	GGAAAtcccc
PLAU	10	75339896-75341319	226 (-)	0.938	GGAAGcacca	14	17121003-17122374	727 (+)	0.921	cagctGGAAAttccgt
			358 (-)	0.814	ctggccTTGCCtttcc					
			509 (+)	0.877	ggggtTTTTC					

Table 2. Early gene NF-κB prediction and human-mouse promoter mapping. The human genes for Early and Late group were mapped to their mouse homologs using the Human Genome Browser gateway using the Human May 2004 (hg17) assembly (NCBI Build 35 produced by the International Human Genome Sequencing Consortium. located on the UCSC Genome Bioinformatics Site, http://genome.ucsc.edu/index.html) and the Mouse (Mus musculus) assembly (Build 33 by the NCBI). For each gene, –1 kb upstream and the first noncoding exon were analyzed. Sequences were analyzed by matrix similarity scores in TRANSFAC using V$CREL_01, V$NFKB_Q6, V$NFKB_C, V$NFKAPPAB_01, V$NFKAPPAB50_01, and V$NFKAPPAB65_01. Significant matches were identified by filtering matrix scores by minimizing the sum the false positive and negative error rates. For multiple matches on the same sequence, the matrix scores (MM) were ranked, and the highest matrix score was selected. Abbreviations used are: Chr., chromosome, NF-κB, location in nucleotides of NF-κB sequence; +/-, sequence located on plus strand or minus strand, respectively; Sequence, is nucleotide sequence of the predicted binding site.
