# Supplementary material for: A TNF-induced gene expression program under oscillatory NF-κB control
Source: BMC Genomics. 2005 Sep 28;6:137. doi: 10.1186/1471-2164-6-137 (PMC1262712; doi:10.1186/1471-2164-6-137)
Supplement: Additional File 3 — Contains the NF-κB-binding site predictions and human-mouse promoter mapping for the Late genes. [file 1471-2164-6-137-S3.pdf]

Table III. Late gene NF-κB prediction and human-mouse promoter mapping.

Late	Human					Mouse				
Promoter	Chr.	Location (bp)	NFkB	MM	Sequence	Chr.	Location (bp)	NFkB	MM	Sequence
**Cyb5**	18	70110091-70111159	129 (-)	0.871	gtggtaTTTCCtctgt	18	85362396-85363465	497 (-)	0.869	GGAAAccacg
			568 (+)	0.988	caataGGAAActcccg			982 (-)	0.972	GGAATgtccc
			963 (-)	0.861	gtggggTTCCCggcgc					
			981 (-)	0.972	GGAATgtccc					
**ECE1**	1	21362214-21363213	100 (-)	0.856	cagggaTTCCCgcctt	4	136319312-136363170	NF	NA	NA
			859 (-)	0.829	ggggtcTCCCCgggag					
**ICAM1**	19	10241779-10242835	448 (-)	0.851	GAAATgcccg	9	20941067-20942141	174 (-)	0.999	GGAAAccccc
			518 (+)	0.86	ggaggATTCC			431 (-)	0.808	caggacTTTCTcacag
			832 (-)	0.986	GGAAAttccg			828 (-)	0.977	GGAAAttcct
			937 (+)	0.869	cgaggTTTCC			933 (+)	0.869	cgaggTTTCC
**IL27RA**	19	14002560-14003684	138 (+)	0.968	tggccTTTCC	NF	NA	NA	NA	NA
			947 (+)	0.992	GGGACtttcc					
**KLRC2**	12	10479853-10480897	NF	NA	NA	6	130353839-130354838	NF	NA	NA
**NAF-1**	5	150424850-150441744	150 (+)	0.885	gtctaGGAAAtcccag	11	54580586-54603649	268 (+)	0.955	tgggtCTTCC
			218 (-)	0.881	ctggatTTGCCcaccc			588 (+)	0.879	ggagaTTTCC
			452 (+)	0.86	gggtcTTTTC			615 (-)	0.981	GGAATccccc
			497 (-)	0.814	ctggagTGCCCtggac			913 (+)	0.983	ggggaATTCC
			570 (-)	0.919	ttggcagGTCCCct					
			765 (+)	0.844	tagagGGGAAggccgc					
			914 (-)	0.998	ggggacTTTCCcaggg					
			953 (+)	0.94	cggcaCTTCC					
**NFkB2**	10	104143320-104145706	60 (+)	0.828	cgtggTGAAAcccca	19	45645210-45646595	80 (+)	0.973	cgggaATTCC
			616 (+)	0.991	GGGGAtcccc			109 (+)	1	ggggcTTTCC
			675 (-)	0.957	GGAAGctccc			715 (+)	0.931	gagggGGAAActcctc
			898 (-)	0.977	tgggaaTTCCCccctc			793 (-)	0.95	GGAAGaaccc
			928 (+)	1	ggggcTTTCC			842 (-)	0.972	GGAATgtccc
								890 (+)	0.863	ctggcTGAAAatccca
								989 (-)	0.923	GGAACcgcct
								1088 (+)	0.992	GGGACtttcc

Table III, con't

**NK4**	16	3054660-3055813	142 (+)	0.939	tggtaCTTCC	NF	NA	NA	NA	NA
			645 (+)	0.947	caGGGAGtttcccc					
			677 (+)	0.977	tggacTTTCC					
**PTGES**	9	129594846-129595898	375 (-)	0.948	GGAAGggcc	2	30863242-30864320	199 (-)	0.936	GGAAGggcca
			459 (-)	0.868	GAAAAgtccc					
			799 (-)	0.867	gaggatTTGCCtggaa					
			985 (+)	0.866	tgtgcTTTCC					
**TAP1**	6	32929572-32930601	583 (-)	0.962	ggtaaGTCCC	NF	NA	NA	NA	NA
**TRAF1**	9	120765569-120769727	1328 (+)	1	ggggaTTTCC	2	34918072-34922576	280 (+)	0.844	tacacGGGAAgacctt
			1385 (+)	0.965	gggtaATTCC			1177 (+)	0.913	accctGGAGAttccca
			1743 (+)	0.852	cagagGGGAAgacctc			1181 (-)	0.945	tggagaTTCCCa
**TRAF3**	14	102312569-102406291	255 (-)	0.86	GGAAAgtgcc	12	105963380-105982148	NF	NA	NA
			938 (+)	1	ggggaTTTCC					
			959 (-)	0.924	GGAAGtgcct					
			1050 (+)	0.996	cGGGACtttcca					
**TRIM16**	17	18544451-18545450	NF	NA	NA	11	62432053-62433052	659 (-)	0.923	GGAAGagcct

Table III. Late gene NF-κB prediction and human-mouse promoter mapping. Same as in Table II for the Late gene promoters.
